# Microfiltration Membranes Modified with Zinc by Plasma Treatment

**DOI:** 10.3390/membranes13040387

**Published:** 2023-03-28

**Authors:** Joanna Kacprzyńska-Gołacka, Monika Łożyńska, Wioletta Barszcz, Sylwia Sowa, Piotr Wieciński

**Affiliations:** 1 Łukasiewicz Research Network—Institute for Sustainable Technologies, 6/10 Pułaskiego St., 26-600 Radom, Poland; 2 Faculty of Chemistry, Warsaw University of Technology, 3 Noakowskiego St., 00-664 Warsaw, Poland

**Keywords:** MS-PVD method, Zn coating, ZnO coating, Zn/ZnO coating, antibacterial properties, polyamide membranes

## Abstract

Polymer membranes play an important role in various filtration processes. The modification of a polyamide membrane surface by one-component Zn and ZnO coatings and two-component Zn/ZnO coatings is presented in this work. The technological parameters of the Magnetron Sputtering-Physical Vapor Deposition method (MS-PVD) for the coatings deposition process show an impact on the influence on the membrane’s surface structure, chemical composition, and functional properties. The characterization of surface structure and morphology were analyzed by scanning electron microscopy. In addition, surface roughness and wettability measurements were also made. For checking the antibacterial activity, the two representative strains of bacteria *Escherichia coli* (Gram-negative) and *Staphylococcus aureus* (Gram-positive) were used. The filtration tests showed that polyamide membranes covered with three types of coatings, one-component Zn coatings, ZnO coatings, and two-component Zn/ZnO coatings, presented similar properties. The obtained results show that using the MS-PVD method for modification of the membrane’s surface is a very promising perspective in the prevention of biofouling.

## 1. Introduction

The freshwater resources in the world are decreasing every year [1,2]. World economies are struggling with the increasing amount of sewage and municipal waste, which poses a threat not only to humans (the specter of lack of drinking water), but also to the preservation of relatively good biodiversity. In accordance with the 6th goal of the Sustainable Development 2030 Agenda, which is to ensure the availability and sustainable management of water and sanitation for all, it seems necessary to develop new technical solutions to recover water from various sources [3]. For this purpose, pressure filtration methods are most often used, which are equipped with membranes to allow the purification of wastewater from metals, organic parts, and microorganisms. A commonly used type of pressure filtration is the microfiltration process. It is mainly used as a prefiltration process to separate as many organic and biological particles as possible from the filtered medium. The efficiency of pre-treatment using microfiltration decreases with the processing time [4,5,6]. This is due to the deposition of organic matter on the surface of the microfiltration membrane, which clogs the membrane pores and prevents separation. The resulting sediment is a favorable place for various microorganisms found in the filtration medium [4,7]. The most common microorganisms in the purified water environment are *Escherichia coli*, *Staphylococcus*, *Enterococcus*, and *Streptococcus* bacteria [8,9]. The development of these microorganisms on the surface of the microfiltration membrane causes a phenomenon called biofouling. This process can be divided into three main stages: attachment, propagation, and biofilm formation [10,11].

The first step consists of the deposition and physical adsorption of microorganisms on the surface of the membrane. The intensity of this process depends on the type of material from which the membrane is made and its physicochemical parameters such as surface charge, hydrophobicity, and roughness [12]. Changing these parameters makes it possible to limit the phenomenon of biofouling at the beginning of its creation. The most popular method of changing the physicochemical parameters of membranes is their surface modification [2,13,14,15,16]. By changing the hydrophilicity and roughness of the membrane, we can significantly reduce the process of attracting and attaching impurities to its surface [17,18,19,20]. Another action preventing the formation of biofouling is the deactivation of bacteria in contact with the surface of the membrane by giving it antimicrobial properties [21,22,23,24]. Both the modification of the physicochemical properties of membranes and giving them bactericidal properties are effective ways to reduce the phenomenon of biofouling. The search for new material solutions enabling the modification of membranes will significantly improve the efficiency of the membrane filtration process. 

One of the effective methods of membrane surface modification is the use of plasma gas (O_2_, Ar, N_2_, and CO_2_), atmospheric plasma, or atomic layer deposition (ALD), and recently developed related methods, such as sequential infiltration synthesis (SIS), offer a tremendously diverse library of chemistries for interface functionalization. This method makes it possible to activate the surface of the membranes and modify their antifouling properties and reduce the tendency to organic fouling [25,26,27]. Recently, a new trend has emerged in the use of PVD plasma surface engineering techniques to modify the surface properties of polymeric materials [28,29,30]. It includes among others, the use of magnetron sputtering technology (MS-PVS) to produce a thin functional coating on the surface of the polymer. The magnetron technology enables very wide possibilities in the production of nanolayers and nanocoatings based on various types of metals and their compounds (e.g., MeO oxides) [31,32,33]. As a result, this method is able to successfully replace the surface modification processes of membranes using nanoparticles. Zinc oxide nanoparticles exhibit toxic properties against various types of bacteria, both from the Gram-positive and Gram-negative groups: *Escherichia*, *Bacillus*, *Pseudomonas*, *Staphylococcus*, and *Enterococcus* [34,35,36,37,38]. According to the authors, the use of magnetron sputtering technology will allow the production of the zinc-oxide-based coating with properties similar to those of ZnO nanoparticles. The deposition of this coating on the surface of the membranes make it possible to impart bactericidal properties. Additional biocidal mechanisms can be unlocked by doping zinc oxide with metals. The metal–metal oxide nanocomposite can significantly enhance the mechanism of oxidative stress due to the efficient photogeneration of highly reactive oxygen species (ROS) [9]. By using a properly designed coating of metal-doped metal oxides, it is possible to impart antibiofouling properties in polymer membranes.

The main aim of this work was to select appropriate parameters of the MS-PVD process for the deposition of one-component Zn and ZnO coatings and two-component Zn/ZnO coatings on the surface of polyamide membranes in order to obtain antibacterial activity. The polyamide membranes with Zn, ZnO, and Zn/ZnO coatings were compared with native (non-coated) membrane surfaces and characterized in terms of structure, morphology, roughness, wettability, stability, and filtration properties. The bactericidal properties of the membranes modified by Zn, ZnO, and Zn/ZnO coatings were also investigated, while maintaining their filtration properties.

## 2. Materials and Methods

### 2.1. Plasma Coating Deposition

The Zn, ZnO, and Zn/ZnO coatings were deposited on membrane filters with a diameter of 4.7 µm (0.22 µm, polyamide, GSV) and a disc of solid polyamide with a diameter of 2.54 cm by the magnetron sputtering method (MS-PVD). This technology belongs to the group of physical vapor deposition methods, in which the substrates of the deposited coating material are obtained as a result of physical phenomena, and the deposition processes are carried out under reduced pressure in the range of 10–10^5^ Pa. The magnetron sputtering method uses a glow discharge in a two-electrode system. The resulting ions are accelerated towards the cathode and cause atoms or particles to be knocked out of the surface of the sputtered electrode (target) and deposited on the surface of the substrate. For the production of pure Zn and ZnO coatings, magnetron power sources equipped with a target (Kurt J. Lesker Company, St. Leonards-on-Sea, UK) made of metallic zinc (Zn 99.999% pure) or zinc oxide (ZnO 99.999% pure), respectively, were used. A two-magnetron system with a common deposition zone equipped with zinc and zinc oxide targets was used to implement the processes of deposition of mixed Zn + ZnO coatings. The diameter of the used target was about 100 mm. The distance between the sample and the plasma source (target) was about 15 cm. During the deposition process, the coatings were created at room temperature in an argon gas atmosphere with a purity 6.0. The Zn, ZnO, and Zn/ZnO coatings were deposited by the changes in the magnetron power source in the range of P_M-Zn_ = 250–300 W and P_M-ZnO_ = 250–300 W. The technological process lasted 60 s using Standard 3 designed and made by the Łukasiewicz Research Network—Institute for Sustainable Technologies (Radom, Poland) and it was without substrate polarization. 

### 2.2. Characterization of Surface Morphology and Structure of Zn, ZnO, and Zn/ZnO Coatings

The SEM observation was performed with the use of a high-resolution scanning electron microscope Helios G5 PFIB CX (ThermoFisher Scientific, Waltham, MA, USA) equipped with a Schottky field emission gun with UC + monochromator (ThermoFisher Scientific, Waltham, MA, USA), in-lens and in-column SE/BSE detectors, and an Octane Elite Super (70 mm^2^) EDS detector (Edax, Pleasanton, CA, USA). 

For measurement of the roughness in the micro-scale, the Form Talysurf PGI 830 Profilograph by Taylor Hobson (Radom, Poland) was used and equipped with a measuring head with a resolution of 8.0 nm. The measurement was carried out using a laser sensor, which moves along the tested sample. The maximum number of collected points is 1,600,000. The measuring speed range was from 0.1 to 2 mm/s. The profilograph has software that enables both 2D (profiles) and 3D (topography) analysis, and also allows the determination of roughness and waviness parameters. During the tests, three roughness parameters were measured: the arithmetic mean deviation of the roughness profile Ra [µm], the arithmetic average of the absolute values of the height peaks in the roughness profile, and the depth of the five lowest depressions of the roughness profile in the interval of the elementary section R_z_ [µm] and the total height of the roughness profile R_t_ [µm]. For each of the tested surfaces, three measurements were made, and then the average values of these measurements were determined. 

### 2.3. Antimicrobial Test 

The antimicrobial properties of the modified polymer membranes were determined using the vacuum filtration method. The antibacterial activity was determined against two strains of bacteria, representing Gram-negative (*Escherichia coli*) and Gram-positive (*Staphylococcus aureus*). Before the tests, the membranes were sterilized with a UV-C lamp in a laminar chamber for 30 min. The inoculum with a starting concentration of (1.5–3.0) × 10^5^ CFU/mL was prepared in saline buffer (KH_2_PO_4_) from a 24 h cell culture. Subsequently, the obtained inoculum was diluted in such a way as to obtain a countable number of colonies on the membranes. The obtained cell suspension was inoculated with 1000 mL of saline buffer (KH_2_PO_4_), from which 10 mL was taken and filtered through the membranes at a pressure of 500 mbar. After filtration, the membranes were placed on TSA (Tryptic Soy Agar) plates and incubated for 24 h at 37 °C. After this time, the grown colonies were counted and the results are expressed as a percentage reduction in cell viability by the formula:

(1) $$R=\frac{I_{w}-I_{p}}{I_{w}}\times 100\;\%$$

where

*R*—percentage reduction in cell viability [%];
*I_w_*—inoculum concentration for the non-coated membrane [CFU/mL];
*I_p_*—inoculum concentration for the coated membranes [CFU/mL].

The research methodology used to determine the antibacterial properties of the coatings deposited by the PVD method was developed for the purpose of testing this type of material. Filtering membranes, operating in a continuous system during filtration of a medium contaminated with microorganism cells, are exposed to their uncontrolled growth and clogging of the membrane pores. The methodological assumptions of this technique result from the most possible representation of the operation of the membrane in real conditions, and hence the use of vacuum filtration and the assessment of antibacterial properties after 24 h. The reference sample was an unmodified (non-coated) membrane. The tests were carried out in three repetitions.

### 2.4. Wettability

The static sessile drop method was used for the investigation of the wettability of Zn-, ZnO-, and Zn/ZnO-modified surfaces. This method is based on the measurement of the contact angle with demineralized water (drop volume of dH_2_O −1 µL). The static contact angle values were automatically examined by a goniometer constructed by Łukasiewicz-ITeE (Radom, Poland). The drop shape analysis is made according to Young’s equation [39]. For the tests, the solid samples of polyamide covered by Zn, ZnO, and Zn/ZnO coatings were used. For each sample, 10 measurements of contact angle were taken. The mean value was calculated after measurements. To increase the speed, accuracy, and precision of this method, computer image processing was used. The computer software is based on the numerical solution of the Young–Laplace equation for the capillary:

(2) $$\Delta \rho =\sigma \left(\frac{1}{R_{1}}+\frac{1}{R_{2}}\right)$$

where

∆*ρ*—the difference between the density of the drop and environment;
*σ*—interfacial tension;
*R*_1_, *R*_2_—principal radii of curvature.

The digital camera investigates the parameters of the drop (diameter, height, etc.). The parameters depend on the angle which forms a drop with the surface. The obtained results should be compared with the so-called dimensionless (theoretical) profiles, which are solutions of the Young–Laplace equation. In this procedure, the surface or interfacial tension is determined from the formula:

(3) $$\sigma =\frac{\Delta \rho gR_{o}^{2}}{\beta }$$

where

*σ*—interfacial tension;
*R*_o_—the radius of curvature of the drop at the top;
*β*—drop shape parameter;
∆*ρ*—the difference between the density of the drop and environment;
*g*—standard gravity.

### 2.5. Filtration Properties and Stability of Coatings

The membrane surfaces, which were modified by three types of coatings, one-component Zn and ZnO coatings and two-component Zn/ZnO coatings, were investigated in view of filtration properties. A non-coated membrane was chosen as a reference sample. By analyzing the volumetric permeate flux, the filtration tests were done. The permeate flux was calculated based on the time needed to filter 50 mL of deionized water through an 8 cm^2^ membrane at a pressure of 0.5 bar.

In addition, the stability of all obtained Zn, ZnO, and Zn/ZnO coatings was also examined. These studies were carried out using a vacuum filtration kit. The process was carried out at a pressure of 0.5 bar. A total of 1 L of deionized water was filtered through the coated membranes in 10 portions of 100 mL each. The test samples were collected after each filtered portion of H_2_O. Collected samples of filtered water were tested for Zn concentration. For this purpose, the samples were first mineralized at 160 °C for 40 min after adding 5 mL of concentrated nitric acid (V). In the mineralizates, which were prepared in this way, the concentration of Zn was analyzed using the flame atomic absorption spectrometer (FAAS) iCE3500 by Thermo Scientific company (Thermo Fisher Scientific, Waltham, MA, USA). The zinc standard solution (1000 µg/mL, Inorganic Ventures, Christiansburg, VA, USA) was used to determine the calibration curve. After the stability tests, the membranes covered with the tested coatings were analyzed in terms of their chemical composition. The tests were performed using the EDS technique by the Octane Elite Super (70 mm^2^) EDS detector (Edax, Pleasanton, CA, USA).

## 3. Results

### 3.1. Morphology and Structure Characterization

**Figure 1:**
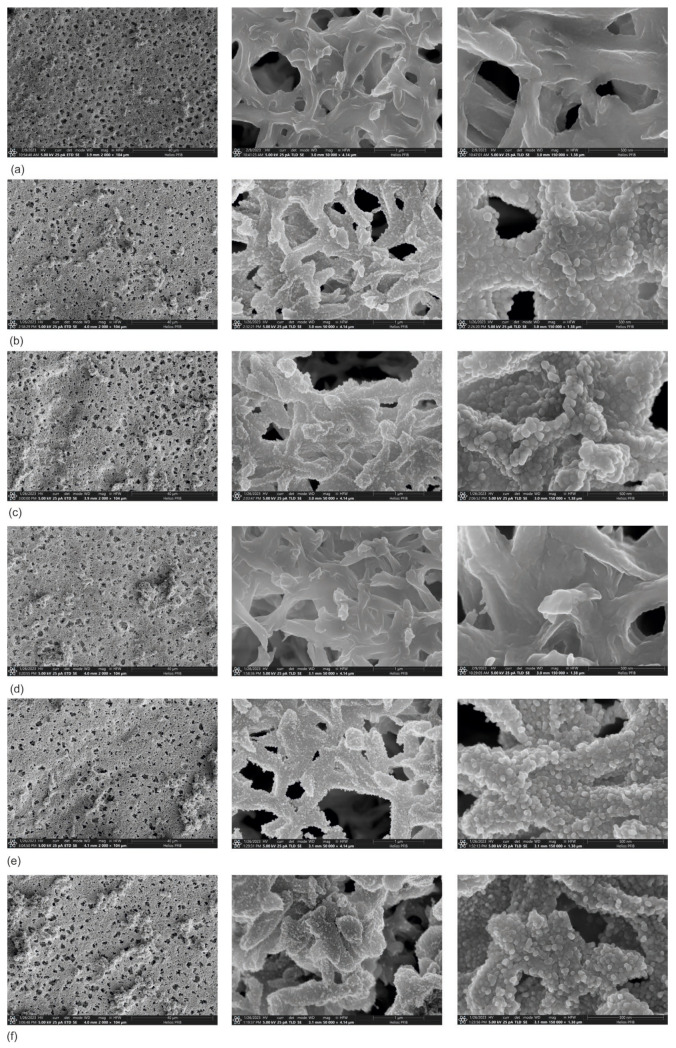
SEM images of the non-coated membrane (**a**) and with Zn, ZnO, and Zn/ZnO coatings deposited at different magnetron power sources (PM): (**b**) P_M-Zn_ = 250 W, (**c**) P_M-Zn_ = 300 W, (**d**) P_M-ZnO_ = 250 W, (**e**) P_M-Zn_ = 250 and P_M-ZnO_ = 250 W, and (**f**) P_M-Zn_ = 300 and P_M-ZnO_ = 250 W.

The observations of membrane surfaces covered with three types of coatings, Zn, ZnO, and Zn/ZnO, are presented in Figure 1. The concluded observations confirmed that it is possible to deposit thin coatings on the surface of the membranes without disturbing their porous structure. All analyzed membranes are characterized by similar proportions and sizes of pores. The type of deposited coating does not change the thickness of the individual fibers of the membrane. The method used allowed for a single application of the coating material over the entire surface of the membrane. It can also be observed that the coating is deposited mainly on the membrane’s surface. Only individual particles of the coatings are visible on the fibers located deeper in the structure.

The conducted SEM observations did not show any significant differences in the porous structure of the membranes covered with coatings compared with the membrane without the coating (Figure 1). The analysis showed that the Zn (Figure 1b,c) and Zn/ZnO coatings (Figure 1e,f) are made of spherical nanoparticles. The diameter of these nanoparticles in Zn coatings is 50–75 nm. The Zn/ZnO coatings are made of smaller nanoparticles with a diameter of about 30–40 nm. The observed structure of the coatings is characteristic of coatings with an island growth mechanism, e.g., for silver-based coatings deposited on polymeric substrates [40,41]. As a result of small surface diffusion, the atoms cannot change their position on the surface, which makes it difficult to grow a continuous shell. Separate islands of material from on the surface as a result of randomly locally growing coating fragments. In the case of high surface diffusion, atoms can easily move across the surface. 

In an effort to minimize energy, the coating material grows more regularly, creating a more continuous coating. In this situation, a dense network of nuclei is formed on the substrate additionally; the nuclei merge with each other and thus the coating grows layer by layer. Such a mechanism is observed in the pure ZnO coating (Figure 1d). 

**Figure 2:**
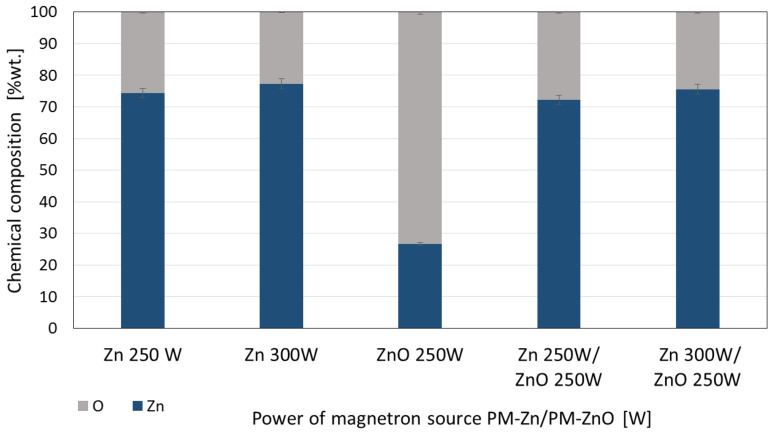
The comparison of mass weight percentage elements (% wt.) for three types of coatings: one-component Zn and ZnO coatings and two-component Zn/ZnO coatings deposited by different magnetron power sources.

The analysis of the chemical composition of the membranes covered with the coatings showed that the coatings of Zn, ZnO, and Zn/ZnO are characterized by very similar chemical compositions. This is probably due to a much higher proportion of pure Zn in the coating structure (Figure 2). The processes of deposition of these coatings were carried out from two simultaneously operating magnetron power sources, one with a metallic pure Zn target and the other with a ZnO target. Pure Zn has a much higher sputtering rate than ZnO. The chemical composition of coating, which was deposited in the described technological configuration, consists mainly of Zn with a small addition of ZnOHence, these coatings have very similar structures and chemical compositions.

**Figure 3:**
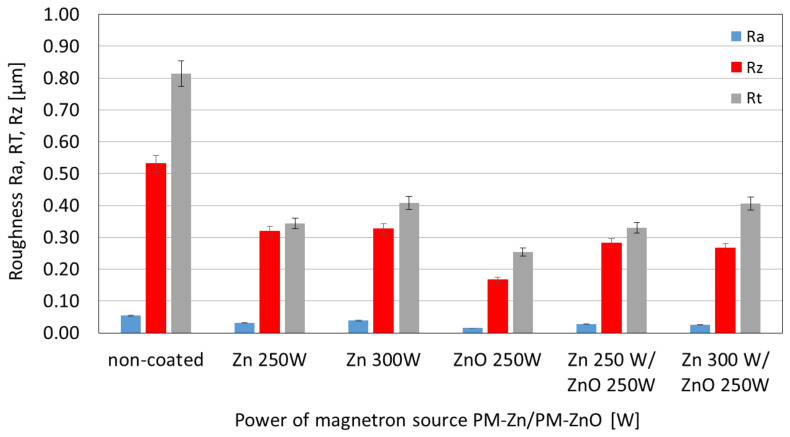
The comparison of the roughness for three types of coatings: one-component Zn and ZnO coatings and two-component Zn/ZnO coatings deposited by different magnetron power sources.

The performed surface roughness analyses on the micro scale are shown in Figure 3. The analyses included a comparison of the Ra, Rz, and Rt values of samples made of pure polymer and samples coated with Zn, ZnO, and Zn/ZnO coatings. 

All samples with coatings were characterized by significantly lower roughness compared with the unmodified (non-coated) polymers. For polymer with ZnO coating, the lowes arithmetic mean profile deviated from the mean line-Ra was obtained. Polymers with a Zn coating were characterized by a higher Ra value, which additionally increased with the increase in magnetron power source. The obtained results confirmed that by depositing a thin coating on the polymer surface, we can significantly reduce the surface roughness, which should have an impact on limiting the phenomenon of bacterial attachment to the substrate and thus limiting the phenomenon of biofouling.

### 3.2. Antibacterial Properties of Zn-, ZnO-, and Zn/ZnO-Coated Membranes

**Figure 4:**
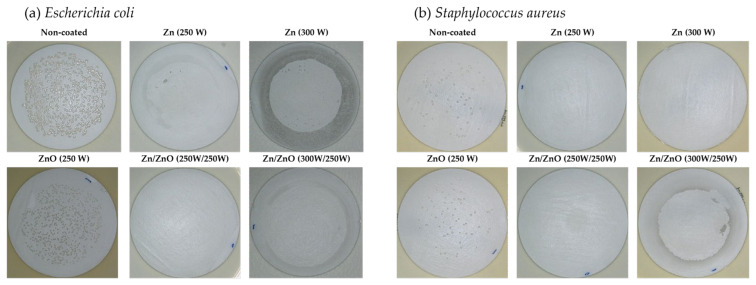
The results of 3D microscope images obtained for antibacterial activity of one-component and two-component coatings against bacteria: (**a**) *Escherichia coli*; (**b**) *Staphylococcus aureus*.

Tests of the antibacterial properties of Zn, ZnO, and Zn/ZnO coatings deposited on membranes using magnetron sputtering were determined against *Escherichia coli* (*E. coli*) and *Staphylococcus aureus* (*S. aureus*). The non-coated membrane was used as a reference material. Figure 4 presents photos from a 3D microscope showing the antibacterial effectiveness of the tested coatings. A similar effect on individual strains of bacteria is noticeable. For both *E.coli* and *S. aureus*, no significant effect on their survival was noted for the ZnO coating with a magnetron power source of P_M_ = 250 W. Other modifications limit the growth of both strains of the tested bacteria.

**Figure 5:**
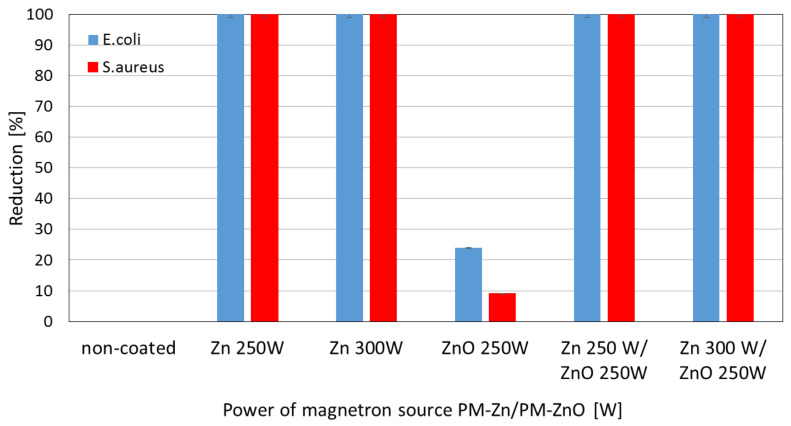
Reduction (%) in the viability of *Escherichia coli* and *Staphylococcus aureus* obtained for Zn and ZnO one-component coatings and Zn/ZnO two-component coatings deposited at different magnetron power sources (P_M_).

As part of the research on the antibacterial properties of one- and two-component coatings, the degree of reduction in the survival rate of the tested bacterial strains was determined. Figure 5 shows the ability to limit the growth of bacteria by the membrane without coating and membranes with tested coatings. It is assumed that a reduction of ≥99.9% within 18–24 h indicates a bactericidal effect in this type of microbiological test. On the other hand, bacteriostatic action is one where a potentially killing agent limits the growth of microorganisms in some way [42]. The analysis of the obtained results indicates the varied performance of the tested membranes without and with coatings. The bactericidal effect was obtained for one-component coatings of Zn deposited with a magnetron power source of 250 W and 300 W. Only a slight bacteriostatic effect was obtained in relation to *E. coli* (24%) and an insignificant effect was obtained for *S. aureus* (9%) for the one-component ZnO coatings deposited with a magnetron power source of 250 W. Figure 5 also presented, that the non-coated membrane did not show a reduction in bacteria viability.did not show a reduction in bacteria viability.

The results obtained for two-component coatings indicate that the bactericidal effect against both bacteria was achieved for all Zn/ZnO coatings (Figure 5). Many studies indicated that ZnO nanoparticles have an antibacterial effect against strains such as: *Escherichia coli* and *Staphylococcus aureus* [43,44,45]. The antibacterial effect of ZnO nanoparticles is related to many parameters, i.e., concentration, morphology, or composition [46]. The nanoparticles exhibit significant antibacterial activity as they have a high surface area to volume ratio which increases as their diameter decreases. The lack of bactericidal effect of the ZnO coating on the microfiltration membrane is most likely due to its structure. The ZnO coating deposited by the magnetron sputtering method is characterized by smooth morphology and consists of a uniform structure where the presence of ZnO nanoparticles was not found. The analysis of the elemental composition of the deposited coatings showed that in the two-component Zn/ZnO coatings, the Zn concentrations dominated, in which a diameter of a particle was about 30–40 nm. Therefore, it was assumed that Zn nanoparticles are responsible for the bactericidal effect of the produced coatings. The mechanism of action of metal nanoparticles on the microbial cells proceeds in different ways. The beginning of the cell destruction process involves the attachment of the nanoparticle to the bacterial membrane through various interactions: electrostatic, van der Waals forces, hydrophobic, or receptor–ligand [47]. In the next step, nanoparticles can penetrate the cell membrane and then block the most important metabolic pathways and, consequently, lead to cell death. The bactericidal effect of metallic nanoparticles consists in inhibiting the activity of enzymes, deactivating proteins, and causing oxidative stress or changes in the process of gene expression in the genetic material [48].

Based on the obtained results, it can be concluded that the use of a one-component Zn coating modified by the plasma method is enough for a bactericidal effect.

### 3.3. Wettability of Zn-, ZnO-, and Zn/ZnO-Coated Membranes

**Figure 6:**
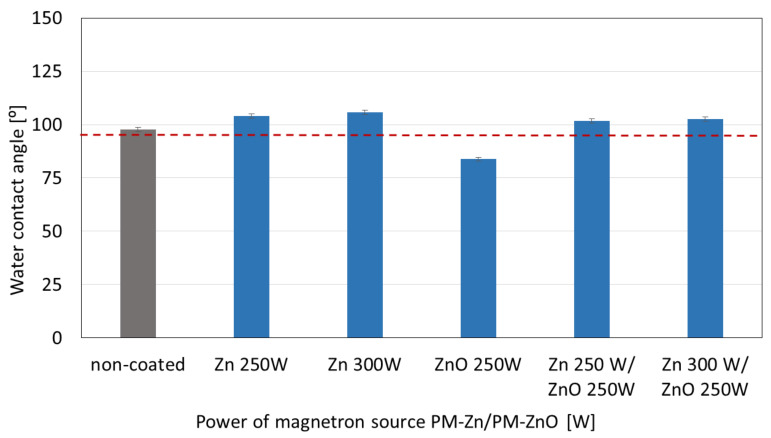
The results of the water contact angle measurements for one-component Zn and ZnO coatings and two-component Zn/ZnO coatings created by different magnetron power sources.

The results of the surface wettability research are presented in Figure 6. The research included a comparison of the water contact angle with demineralized water of samples made of pure polymer and samples covered with Zn, ZnO, and Zn/ZnO coatings. 

The water contact angle for the pure polymer was 97.8° ± 1.1°. Only for the ZnO coatings, a decrease in the contact angle to 83.9° ± 1.8 was observed. This proves that it was enabled with hydrophilic properties compared with the non-coated sample. For Zn coatings, which were deposited at the magnetron power sources of 250 W and 300 W, increases in the water contact angle of 6.3% and 8.1% were observed, respectively. ZnO addition in the two-component coatings caused only a slight decrease in the water contact angle values compared with the one-component coatings.

### 3.4. Filtration Properties and Stability of Zn-, ZnO-, and Zn/ZnO-Coated Membranes

**Figure 7:**
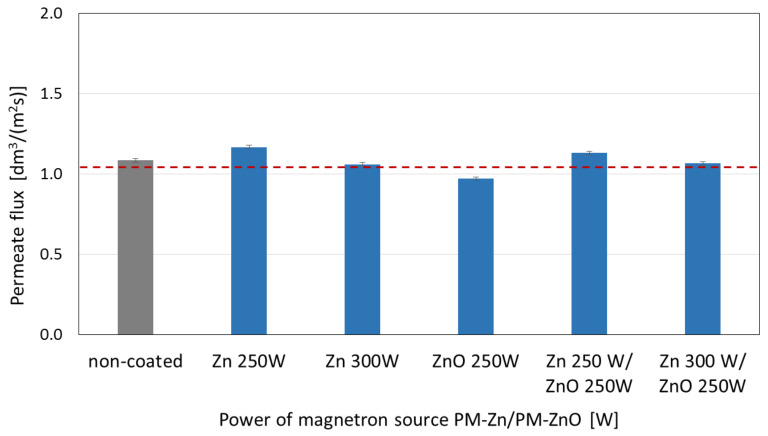
The effect of changing the magnetron power source for one-component Zn and ZnO coatings and two-component Zn/ZnO coatings covered the membranes’ surfaces on the permeate flux determined during filtration.

The membranes’ surfaces coated with Zn and ZnO coatings and two-component Zn/ZnO coatings were also tested for the value of permeate volumetric flux determined during the filtration of demineralized water through the membranes (Figure 7).

The highest values of permeate flux were obtained for the membrane with the Zn coating deposited at the magnetron power source of 250 W. The increase in the magnetron power source to 300 W caused a drop in the permeate flux to the level obtained for the non-coated membrane. ZnO addition in two-component coatings did not significantly affect the permeate flux obtained for the one-component coatings.

**Figure 8:**
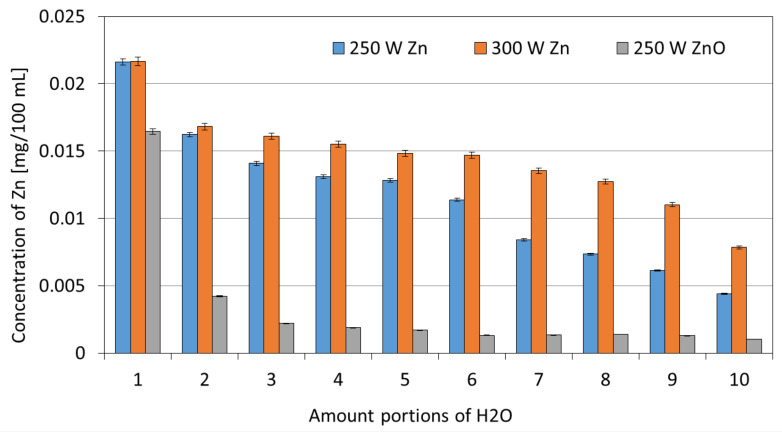
The Zn concentrations in filtrates obtained in the filtration processes of demineralized water through membranes with one-component coatings.

**Figure 9:**
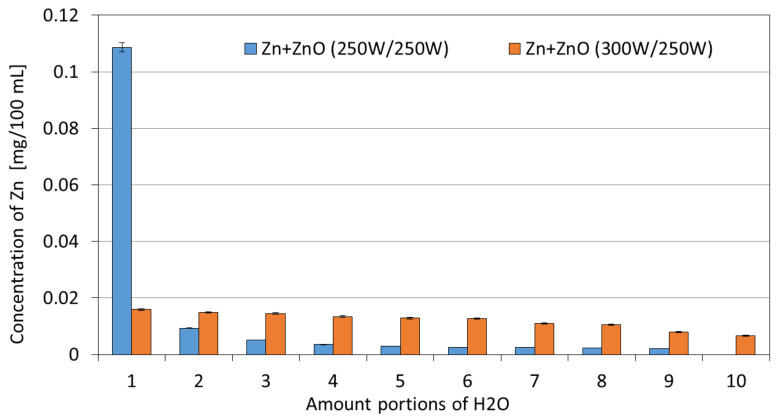
The Zn concentrations in filtrates obtained in the filtration processes of demineralized water through membranes with two-component coatings.

The obtained coatings were also tested for their stability in water. The collected filtrates were mineralized and analyzed for Zn concentrations. The obtained results are shown in Figure 8 and Figure 9.

In the case of the one-component Zn coatings, a similar scheme of leaching in the water can be observed. The Zn concentration decreases with each filtered portion of water. For the Zn coating deposited at 300 W, these concentrations are higher compared with the Zn coating deposited at a magnetron power source of 250 W. This is probably due to the higher amount of Zn contained in the coating with a magnetron power source of 300 W. Only in the first 100 mL of water, in the case of the ZnO coating, an increased concentration of Zn can be observed, similar to the values obtained for the Zn coatings. In subsequent portions of water, the concentration of Zn is similar and reaches low values within the range of 0.002–0.004 mg for every 100 mL. 

In the case of two-component Zn/ZnO coatings, a different pattern of Zn leaching was observed. For the Zn/ZnO coating deposited at the magnetron power source of 250/250 W, the increased concentrations of ions were observed only in the initial phase of the process (for a volume of 100 mL). This phenomenon may be due to the leaching of particles not bound to the deposited coating. In subsequent portions of water, the concentration of Zn can be described as low and not exceeding the content of 0.01 mg Zn/100 mL. However, for the two-component Zn/ZnO (P_M_ = 300 W/250 W) coatings, the Zn concentrations remain at a similar level with a downward trend along with the duration of the leaching process from the initial Zn concentration of 0.016 mg for the first portion of H_2_O to the final concentration of 0.007 mg Zn for the last portion of H_2_O. The values of the total Zn concentration after filtering 1 L of deionized water are similar for almost all tested one-and two-component coatings excluding the ZnO coating—in the range of 0.12–0.14 mg/L. In the case of the ZnO coating (P_M_ = 250 W), the total Zn concentration was several times lower and amounted to 0.03 mg/L. The obtained relatively low Zn concentrations in the filtrates confirm that the membranes with coatings are characterized by high stability in water conditions.

**Figure 10:**
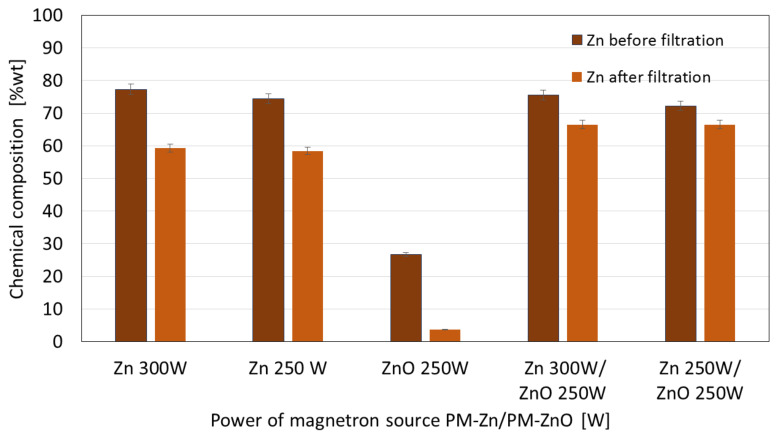
The comparison of the chemical composition (% wt.) before and after filtration for one-component Zn and ZnO coatings and two-component Zn/ZnO coatings deposited by a different magnetron power sources.

The membranes, after stability tests in deionized water, were subjected to elemental composition analysis using the EDS technique. The obtained results were compared with the results for the membranes before the filtration process. The results of the element compositions (% wt.) before and after the filtration process for the one-component Zn and ZnO coatings and the two-component Zn/ZnO coatings deposited at different magnetron power sources are shown in Figure 10.

The results obtained before filtration indicated that the Zn concentration in the one-component Zn coatings increased with the increase in the magnetron power source from 250 W to 300 W. A similar observation was made for the membranes with Zn/ZnO coatings before the filtration process. The Zn mass weight percentage in presented coatings was increased with an increase in the magnetron power source from 250 to 300 W. For both cases, one-component Zn coatings (P_M_ = 250 W and 300 W) and two-component ZnO coatings (P_M_ = 250/250 W and 300/250 W), we observed that after the filtration process, the Zn concentration was decreased. The decrease in the Zn concentration after the filtration process of the Zn coatings is higher than in the case of the Zn/ZnO coatings. This suggests that the addition of ZnO improves the stability of the coatings.

For the one-component ZnO coating, the lowest Zn mass weight percentage before and after the filtration process was found in the comparison with the other coatings. This is probably due to the lower rate of ZnO deposition on the membrane surface, which results in the formation of a very thin ZnO coating and, therefore, contains fewer Zn atoms. Coatings obtained by PVD methods are adhesive coatings, which means that they are not chemically bound to the substrate; therefore, the chemical stability of membranes with coatings is related to their adhesion and cohesion. The conducted works indicated a higher degree of release of zinc ions in the initial phase of the filtration process, which is related to the cohesion of the obtained coatings. In the initial phase of filtration, unbound coating particles are released into the filtered medium. In the further stage of filtration, a decrease in the amount of released ions was observed. The examination of the chemical composition of the membranes after the filtration process confirmed that the coating was not removed from its surface. The obtained results indicate that the authors obtained coatings with sufficient adhesion and cohesion to the substrate. 

## 4. Conclusions 

In this article, a polyamide membrane was coated with three types of coatings: one-component Zn coatings, one-component ZnO coatings, and two-component Zn/ZnO coatings deposited by the MS-PVD method with different magnetron power sources. The research presents the effect of the magnetron power source on the functional properties of plasma-modified membranes. The analysis of the membranes’ structures with the deposited coatings confirmed that it is possible to deposit thin coatings on the membrane surface without disturbing its porous structure. The morphology analysis showed that the Zn and Zn/ZnO coatings are made of spherical nanoparticles. The diameter of these nanoparticles in the Zn coatings is almost twice larger than the diameter of nanoparticles in the Zn/ZnO coatings. The ZnO coating has a completely different structure. We obtained a coating with a homogeneous structure that evenly covers the individual fibers of the membrane. The deposited Zn and Zn/ZnO coatings were tested for antibacterial properties against two representative strains of bacteria belonging to the Gram-positive and Gram-negative groups. The results of the antimicrobial tests confirmed a significant impact of the magnetron power source of the Zn or ZnO target on the biological effectiveness of the obtained membranes’ surfaces. Both one-component Zn coatings and two-component Zn/ZnO coatings were shown to have a bactericidal effect (>99.9% reduction in bacterial cell viability) against *Escherichia coli* and *Staphylococcus aureus*. The membrane covered with the ZnO coating did not show a bactericidal effect, which is probably related to the structure of the coating. The analysis of the elemental composition showed that the deposited two-component coatings contain mostly zinc nanoparticles, and it is to them that the antibacterial effect against the tested strains of bacteria is attributed. The conducted investigation also showed that the membranes without and with coatings were characterized by similar contact angles in the wettability analysis. The authors found that the best filtration properties were indicated for the membranes with Zn/ZnO coatings. Similar to the membranes with one-component coatings, the membranes with the Zn/ZnO coatings were characterized by a similar flow compared with the non-coated membranes. However, the stability of these coatings in the aqueous environment was better. The analysis of the presence of zinc in the filtered medium and the analysis of the chemical composition of the membranes after the filtration process showed that in the case of membranes with two-component coatings, we observed a smaller amount of zinc in the filtrate and a smaller decrease in the amount of zinc on the membrane after filtration. According to the authors, the best properties were obtained for the membrane with a two-component coating Zn/ZnO (300 W/250 W). This membrane is characterized by very good bactericidal properties (Figure 5) and the best stability in the aqueous environment, as evidenced by the stable and low amount of ions released into the permeate in the filtration processes (Figure 9). Other parameters such as wettability and permeability are at the level of the uncoated membrane. The authors confirmed that the application of magnetron sputtering technology is a very good solution enabling the production of functional thin films on the membrane’s surface. The obtained results confirmed that by depositing a thin layer of a Zn and ZnO coating, we can add bactericidal properties to a polymer membrane without disturbing its filtration properties. 

## Data Availability

Not applicable.
